# Sustained low efficiency dialysis should not be interrupted for performing transpulmonary thermodilution measurements

**DOI:** 10.1186/s13613-018-0455-x

**Published:** 2018-11-23

**Authors:** Stefanie Geith, Lynne Stecher, Christian Rabe, Stefan Sack, Florian Eyer

**Affiliations:** 1 Division of Clinical Toxicology and Poison Control Centre Munich, Department of Internal Medicine II, TUM School of Medicine, Technical University of Munich, Munich, Germany; 2 Institute of Medical Informatics, Statistics, and Epidemiology, TUM School of Medicine, Technical University of Munich, Munich, Germany; 3 Department of Cardiology, Pneumology and Intensive Care, Emergency Center for Internal Affairs, Academic General Hospital Munich - Hospital Schwabing, Munich, Germany

**Keywords:** Sustained low efficiency dialysis (SLED), Transpulmonary thermodilution measurement (TPTD), Patients with multiple organ failure (MOF)

## Abstract

**Background:**

Treatment of multiple organ failure frequently requires enhanced hemodynamic monitoring. When renal replacement is indicated, it remains unclear whether transpulmonary thermodilution (TPTD) measurements are influenced by renal replacement therapy (RRT) and whether RRT should be paused for TPTD measurements. Our aim was therefore to investigate the effect of pausing RRT on TPTD results in two dialysis catheter locations.

**Materials and methods:**

In total, 62 TPTD measurements in 24 patients (APACHE: 32 ± 7 [mean ± standard deviation (SD)]) were performed using the PiCCO™ system (Pulsion, Germany). Patients were treated with sustained low efficiency dialysis (SLED; Genius™ system, Fresenius, Germany) as RRT. Measurements were taken during ongoing hemodialysis (HD, HDO), during paused HD (HDP) and immediately after termination of HD and blood restitution (HDT). Dialysis catheters were placed either in the superior vena cava (SVC, 19 times) or in the inferior vena cava (IVC, 5 times). Statistical analysis was performed to assess the effects of the measurement setting, SLED (blood flow rate) and the catheter location, on cardiac index (CI), global end-diastolic volume index (GEDVI) and extravascular lung water index (EVLWI) as measured by TPTD. Multilevel models were used for the analysis due to the triplicate measurements and due to 12 out of 19 SVC and 2 out of 5 IVC patients having more than one TPTD measured.

**Results:**

CI and GEDVI were significantly higher at time point HDP compared to both HDO and HDT. In contrast, values for EVLWI were lower at HDP when compared to HDO and HDT. These findings were independent of the site of dialysis catheter insertion and blood flow rate.

**Conclusions:**

PiCCO™ measurements assessed at paused SLED significantly deviate from ongoing and terminated SLED. Therefore, the dialysis system should not be paused for measurements. TPTD measurements in patients with PiCCO monitoring seem sufficiently reliable during ongoing SLED as well as after its termination. An effect of dialysis catheter location (SVC vs IVC) and blood flow rate on PiCCO™ measurements could not be shown.

## Background

Measurement of key parameters such as the cardiac index (CI) is crucial for appropriate hemodynamic monitoring of critically ill patients. Techniques like indicator dilution are considered as most appropriate and can be obtained either by pulmonary arterial catheter or by transpulmonary thermodilution (TPTD) [1, 2]. Since up to 30% of critically ill patients with multiple organ failure (MOF) develop acute kidney injury (AKI), 10% of them will require renal replacement therapy (RRT) [3]. Thus, simultaneous use of invasive hemodynamic monitoring including pulse contour analyses and TPTD during RRT is common practice. Nowadays, sustained low efficiency dialysis (SLED) for RRT is more frequently used as compared to intermittent RRT because of a better hemodynamic tolerability, but also for economic reasons.

There are concerns about the applicability of indicator dilution techniques including TPTD during RRT [4–8]. As TPTD relies on subtle temperature changes in arterial blood in response to central venous injection of a cold saline bolus, a pulsatile extracorporeal circuit might introduce a measurement bias. In addition, there are other potential confounders of the RRT on thermodilution: loss of indicator in the circuit, changes in blood pump flow and finally an immediate proximity of the central venous catheter (CVC) and dialysis catheter (e.g., both catheters in jugular veins or both catheters in the femoral veins), which might impair TPTD by withdrawing the fluid bolus injected via the CVC via the dialysis catheter [3].

Moreover, there is a controversy regarding the optimal time point for measurement [9]. One current matter of discussion is whether the ideal measurement should be performed during ongoing or at paused RRT. Some authors advocate pausing RRT during measurements [4, 5], while others do not observe any influence of pausing the RRT on TPTD results [10–12]. As in most of these studies continuous RRT methods like CVVH, CVVHD and CVVHF were assessed, only a few directly evaluated the influence of SLED on TPTD measurements. Recently, Huber et al. published a systematic evaluation of the influence of connected or disconnected SLED on TPTD [3]. The effect of paused RRT on the TPTD measurements in patients with SLED has, to the best of our knowledge, not yet been systematically evaluated. Therefore, the aim of this study is to provide data about the influence of paused SLED on TPTD measurements.

## Materials and methods

### Patients

Twenty-four critically ill patients (17 males, 7 females, see Table 1 for group characteristics) with MOF, defined as acute renal failure according to the KDIGO classification in combination with a circulatory failure requiring catecholamine therapy, treated in the medical intensive care units of the Clinic for Cardiology, Pneumology and Internal Intensive Care at the Clinic Munich, Schwabing, and the Department of Clinical Toxicology, Klinikum rechts der Isar, Technical University of Munich, were prospectively enrolled during a 12-month study period. The study protocol was approved by the ethics committee of the Faculty for Medicine of the Technical University of Munich (ethics no. 159/14). The study was performed in accordance with the Declaration of Helsinki and Good Clinical Practice ICH-E6.

**Table 1:** Patient characteristics at baseline during the first dialysis

Position of the dialysis catheter	Cases [mean ± SD]		
	Superior vena cava (SVC)	Inferior vena cava (IVC)	Total
Patients	19	5	24
Age (years)	66 ± 11 (*n* = 18)	59 ± 12 (*n* = 5)	65 ± 11 (*n* = 23)
APACHE score	32 ± 8 (*n* = 16)	31 ± 2 (*n* = 3)	32 ± 7 (*n* = 19)
MAP (mmHg)	73 ± 14 (*n* = 18)	85 ± 20 (*n* = 2)	75 ± 15 (*n* = 20)
Heart rate (1/min)	101 ± 14 (*n* = 18)	93 ± 18 (*n* = 2)	100 ± 14 (*n* = 20)
Central venous pressure (mmHg)	14 ± 5 (*n* = 8)	–	14 ± 5 (*n* = 8)
Blood flow rate (mL/min)	188 ± 34 (*n* = 18)	165 ± 21 (*n* = 2)	186 ± 33 (*n* = 20)
Blood withdrawal (mL/h)	239 ± 135 (*n* = 18)	225 ± 35 (*n* = 2)	238 ± 128 (*n* = 20)

Patients with shock, defined as a mean arterial pressure (MAP) below 60 mmHg after application of 1000 mL crystalloids, received an advanced hemodynamic monitoring and were eligible for enrollment

### Hemodynamic assessment

A 13.5-F dialysis catheter (Niagara 13.5 F × 15/20 cm, C.R. Bard Inc., NJ, USA) was placed in either the SVC or the IVC as confirmed by radiological position monitoring. Blood flow rates varied between 150 and 260 mL/min.

Patients also received a multilumen CVC (Arrow 8.5 F × 20 cm, Teleflex Inc., USA) for infusion of medications with the tip in the SVC.

The cardiac index (CI), global end-diastolic volume index (GEDVI) and extravascular lung water index (EVLWI) were measured for each patient in triplicate in three different settings: (1) measurement while ongoing HD (HDO) (SLED, Genius™ system by Fresenius, Germany); (2) measurement at paused HD (HDP) without disconnection of the system for the duration of the measurements; and (3) measurement performed shortly after RRT was terminated after blood restitution (HDT). For each measurement, a bolus injection of 15 mL cooled normal saline was injected through a CVC placed in the SVC. The triplicate measurements were performed consecutively with the smallest possible delay and immediately after release of each injection, using the same approach and device type [PiCCO-Monitor (PiCCO Pod/Infinity Delta, Dräger, Germany)]. In order to guarantee stable hemodynamic conditions, MAP and heart rate were noted during each measurement series and doses of vasopressors, inotropes and analgosedative agents as well as volume application, and ventilation parameters remained unchanged during measurement, ensuring that the clinical condition of the patient at different measurement time points was in a “steady state.”

### Statistical analysis

Statistical analysis was performed using R version 3.1.0 [13]. To assess intra-individual changes in CI, EVLWI and GEDVI between each of the three measurement settings (HDO, HDP, HDT), multilevel (mixed) models were fitted due to the triplicate measurements and measurements for more than one dialysis sessions for 12 out of 19 SVC and 2 out of 5 IVC patients. All measurements of a triplicate were included in the analysis. The models therefore included a patient random effect and a dialysis within patient random effect. Each measurement setting (HDO, HDP and HDT), together with the catheter location, was included as fixed effects. To assess the potential effect of blood flow rates on CI, EVLWI and GEDVI, blood flow rate was also included as a fixed effect in additional analyses. The estimated coefficients from these models are presented together with 95% confidence intervals. The baseline data are presented as mean ± standard deviation (SD). To assess the differences in variability between each of the measurement settings, the SD was calculated for the triplicates at each setting for each dialysis run. Paired t-tests were used to compare the SD between measurement settings. To explore if the first measurement of the triplicate leads to higher variability, mean absolute differences between the first and second, second and third and first and third measurements were calculated for each measurement setting. The coefficient of variation (CV) was calculated as ${\text{CV}} = {\text{SD/Mean}}$; the coefficient of error (CE) was calculated as ${\text{CE}} = {{\text{CV}} \mathord{\left/ {\vphantom {{\text{CV}} {\sqrt {{\text{number }}\;{\text{of}}\;{\text{boluses}}} }}} \right. \kern-0pt} {\sqrt {{\text{number }}\;{\text{of}}\;{\text{boluses}}} }}$; precision was calculated as ${\text{precision}} = 2*{{\text{CV}} \mathord{\left/ {\vphantom {{\text{CV}} {\sqrt {{\text{number }}\;{\text{of}}\;{\text{boluses}}} }}} \right. \kern-0pt} {\sqrt {{\text{number }}\;{\text{of}}\;{\text{boluses}}} }}$; and the least significant change (LSC) was calculated as ${\text{LSC}} = {\text{CE}}*1.96*\sqrt 2$ [14]. For all analyses, a significance level of 0.05 has been used. Due to the explorative manner of this study, no adjustment for multiple comparisons has been made.

## Results

### Higher variability of TPTD measurements at HDP

**Fig. 1:**
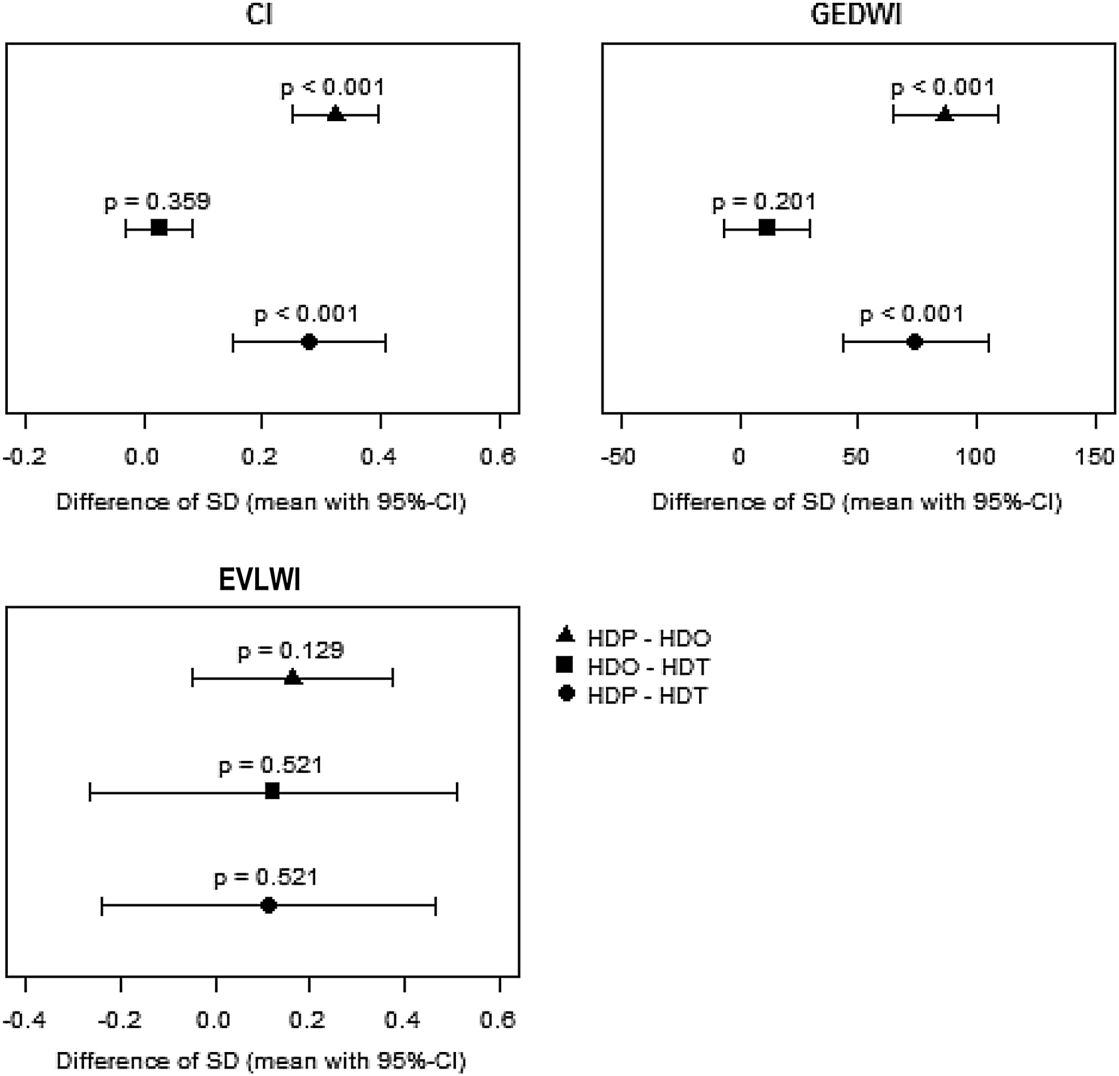
Mean differences in SD for CI, GEDVI, EVLWI. Shown are the differences in SD (with confidence intervals) between the triplicate measurements for the parameters cardiac index (CI) (**a**), global end-diastolic volume index (GEDVI) (**b**) and extravascular lung water index (EVLWI) (**c**) for paused versus during (HDP/HDO) (filled triangle), during versus stopped (HDO/HDT) (filled square) and paused versus stopped HD (HDP/HDT) (filled circle)

A comparison of the single measurements within a triplicate revealed a higher variability of the measurement results and thus a lower precision for CI and GEDVI at HDP compared with HDO or HDT as evidenced by an increased SD; this was not the case for EVLWI measurements (Fig. 1).

Subsequently, the mean differences for CI and GEDVI between the single measurements were calculated, to test if the first measurement after pausing the HD should be rejected. The mean absolute differences between first and second measurements were smaller than the mean absolute differences between the second and third measurements, and therefore, the first measurement after pausing the SLED could not account for the higher variability. The mean difference for the EVLWI showed no significant difference between the settings (Table 2).

**Table 2:** Mean absolute differences between the first, second and third measurements for CI, EVLWI, GEDVI at HDP

	Mean absolute difference between first and second measurements (min, max)	Mean absolute difference between first and third measurements (min, max)	Mean absolute difference between second and third measurements (min, max)
CI	0.58 (0.01, 2.61)	0.67 (0.03, 2.57)	0.63 (0.00, 1.68)
EVLWI	0.79 (0.00, 5.00)	1.12 (0.00, 6.00)	0.89 (0.00, 6.00)
GEDVI	142 (12, 825)	173 (0, 540)	159 (1, 515)

### TPTD measurements and catheter location

**Fig. 2:**
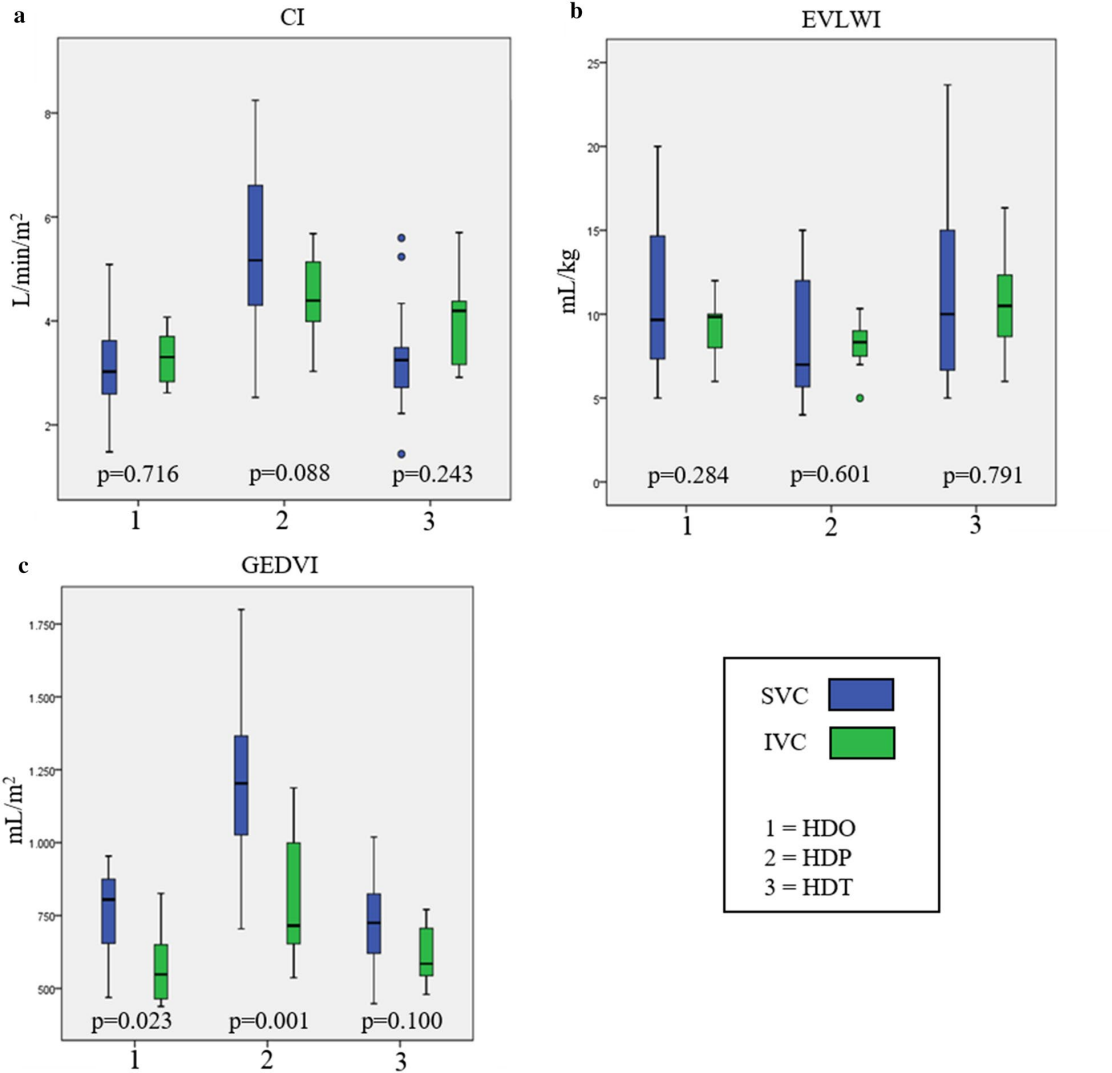
Boxplots CI, EVLWI, GEDVI. Shown are the values (median/25. and 75. percentile) for the parameters cardiac index (CI) (**a**), extravascular lung water index (EVLWI) (**b**) and global end-diastolic volume index (GEDVI) (**c**) at ongoing HD (HDO), paused HD (HDP) and terminated HD (HDT) for measurements via a catheter in the superior vena cava (SVC) or inferior vena cava (IVC). The given *p* values indicate the differences between SVC and IVC

There was no significant evidence of an effect of dialysis catheter location on any of the outcome variables (Fig. 2, Table 3). The mean absolute values for HDO and HDT were in the same range, whereas the results differed from HDP. Figure 2 shows that the mean values were comparable for both locations without neither a significant nor meaningful difference. For example, the CI at HDO in the SVC (CI_SVC_ = 3.08 ± 0.21 l/min/m^2^) was similar to the one measured in the IVC (CI_IVC_ = 3.19 ± 0.22 l/min/m^2^). Comparable results were found for the EVLWI and GEDVI and additionally in the two other settings (HDP, HDT).

**Table 3:** Estimated differences and 95% confidence intervals for CI, GEDVI and EVLWI in each setting between time points and between catheter locations

Outcome measurement	Comparison	Estimated difference (95% CI)	P value
CI (L/min/m^2^)	HDP versus HDO	2.0 (1.9, 2.2)	< 0.001
	HDP versus HDT	1.7 (1.5, 1.9)	< 0.001
	HDT versus HDO	0.4 (0.2, 0.6)	< 0.001
	IVC versus SVC	− 0.1 (− 1.2, 1.0)	0.879
GEDVI (mL/m^2^)	HDP versus HDO	470.0 (431.1, 508.9)	< 0.001
	HDP versus HDT	452.1 (400.0, 504.2)	< 0.001
	HDT versus HDO	17.8 (− 34.0, 69.7)	0.500
	IVC versus SVC	− 207.9 (− 430.3, 14.4)	0.065
EVLWI (mL/kg)	HDP versus HDO	− 2.5 (− 2.9, − 2.2)	< 0.001
	HDP versus HDT	− 3.1 (− 3.6, − 2.6)	< 0.001
	HDT versus HDO	0.5 (0.0, 1.0)	0.038
	IVC versus SVC	− 0.2 (− 3.9, 3.5)	0.904

	HDO (SVC/IVC)	HDP (SVC/IVC)	HDT (SVC/IVC)
*CI*			
Mean	3.08/3.19	5.26/4.46	3.27/3.79
SD	0.21/0.22	0.32/0.34	0.25/0.36
CV [%]	7/7	6/8	8/9
CE [%]	4/4	4/4	4/5
Precision [%]	8/8	7/9	9/11
LSC [%]	11/11	10/12	12/15
*GEDVI*			
Mean	758/606	1286/866	743/641
SD	37/55	87/89	44/43
CV [%]	5/9	7/10	6/7
CE [%]	3/5	4/6	3/4
Precision [%]	6/10	8/12	7/8
LSC [%]	8/15	10/16	10/11
*EVLWI*			
Mean	11/10	8/9	11/11
SD	0.9/0.62	0.63/0.41	1.45/1.2
CV [%]	8/6	8/5	13/11
CE [%]	5/4	5/3	8/6
Precision [%]	9/7	9/5	15/13
LSC [%]	13/10	13/7	21/17

### CI and blood flow rate

The addition of blood flow rate to the multilevel models did not significantly alter any of the measured outcome variables (*p* = 0.784 for CI, *p* = 0.600 for GEDVI, *p* = 0.237 for EVLWI).

### TPTD measurements for HDO, HDP and HDT

A summary of the TPTD measurements for ongoing (HDO), paused (HDP) and terminated (HDT) dialysis is given in Fig. 2. The results comparing the measurements between time points are presented in Table 3. The pausing of SLED led to significantly increased values of CI and GEDVI compared to HDO and HDT and significantly decreased values of EVLWI. Although statistical significance was also reached for the comparisons of CI and EVLWI between HDO and HDT, these differences are not considered to influence the outcome of the TPTD as they are with about 10% below or around the level of LSC (CI_SVC/IVC_ 10–15%, ELWI_SVC/IVC_ 8–21%).

## Discussion

Our study shows that (1) ongoing SLED does not influence TPTD measurements, (2) pausing SLED for TPTD significantly influenced results due to a higher variability of measurements, and (3) results were not influenced by dialysis catheter site (SVC vs IVC) and blood flow rate.

SLED is frequently used instead of other better evaluated continuous RRTs, e.g., CVVH(D/F). We compared TPTD during/at paused SLED to measurements, which were taken under standard conditions after terminated SLED and after blood re-transfusion, regaining a steady state. The results of TPTD measurements during ongoing SLED were comparable to the results of the measurements after terminated SLED, while TPTD measurements assessed at paused SLED significantly deviated from ongoing and terminated SLED.

Although we have detected statistically significant differences in CI and EVLWI between ongoing and terminated SLED in combination with a nonsignificant difference in GEDVI, the clinical impact of these differences is negligible. The precision of the triplicate measurements at ongoing, paused and terminated SLED for CI is ≤ 10% and thus acceptable. For GEDVI and ELWI, some values at paused (GEDVI), respectively, terminated SLED (ELWI) are above this limit and thus less precise. The differences in, e.g., CI between ongoing/terminated and paused SLED are statistically significant, but still under the threshold of 15% [15–17] to be clinically relevant as well as below the threshold of LSC [14]. For differences below the threshold of LSC, it is not sure that the changes are true or linked to the error of the technique [14]. In contrast considering the absolute difference in CI of 0.4 L/min/m^2^ for ongoing versus terminated SLED in clinical practice, this would unlikely cause a change in the existing treatment regime, as opposed to a difference in CI of 2.0 L/min/m^2^ (paused vs ongoing SLED) or 1.7 L/min/m^2^ (paused vs terminated SLED), respectively.

In contrast, pausing SLED for the duration of TPTD measurements significantly influences TPTD measurements presumably by inducing turbulences or temperature differences and fluctuations, but neither dialysis catheter position nor blood flow rate had significant influence on TPTD variables. TPTD is thus considered sufficiently accurate during ongoing SLED as well as after its termination although one would expect higher temperature fluctuations than with continuous RRTs. Our results show that TPTD measurements differ significantly depending upon whether they are obtained during or shortly after terminating SLED compared with pausing SLED, indicating that pausing SLED to perform TPTD measurements results in inaccurate and imprecise estimates of cardiac index and other important hemodynamic variables.

Investigators have found variable effects of continuous renal replacement therapy on TPTD measurements, with different findings depending upon RRT catheter location, flow rates, and whether RRT is ongoing, paused or stopped during the TPTD measurements. Our findings suggest that pausing RRT results in imprecise and inaccurate TPTD-derived hemodynamic measurements, contributing to the existing body of knowledge about the effects of RRT on TPTD hemodynamic measurements. Our study expands existing knowledge because we focus on SLED, whereas previous studies have examined the effects of CVVH(D) on TPTD measurements with controversial results (Table 4).

**Table 4:** Studies investigating the influence of running RRT and/or catheter location on TPTD

Author	No. of patients	RRT	CVC	Dialysis Cath. Loc.	Blood flow (mL/min)	Summary of main findings	Effect dialysis cath. location	Recommendation
Mason et al. [5]	26	CVVH	–	–	–	*Running CVVH*	n.d.	Measurements should be made with CVVH temporarily switched
						Underestimates CO and ITBV		
						Overestimates EVLWI		
						No correlation between CVVH pump speed, fluid exchange rate or use of inotropes/pressors and the changes in cardiovascular parameter on and off CVVH		
Martinez-Simon et al. [4]	1	CVVHD	SVC distal lumen of a three lumen dialysis catheter	SVC	–	*Running RRT*	+*	RRT should be made with paused RRT
						Reduces CI and ITBVI		
Sakka et al. [10]	24	CVVHF	SVC	SVC, IVC	80–150	*Running RRT*	–**	RRT should not be paused
						Associated with a significant, but clinically not relevant decrease in CI and ITBVI		
						No influence on EVLWI		
						No significantly different influence of the dialysis catheter tip position on the changes by RRT in CI, ITBVI and EVLWI		
						As a marker of good acquisition quality, variability of results was more pronounced between patients than between time points		
Neirynck et al. [6]	9	CVVH	SVC	IVC (dialysis catheter before injection site) = “*correct*” Dialysis catheter position *between* the thermodilution injection and detection sites = “*faulty*”	–	*During RRT*	+*	–
						CI and GEDVI decrease		
						EVLWI increases		
Van Craenenbroeck et al. [8]	29	CVVH	SVC	IVC (dialysis catheter before injection site) = “*correct*” Dialysis catheter position *between* the thermodilution injection and detection sites = “*faulty*”	130 ± 35	*During RRT*	+*	“*Ideally, optimal* (hemodynamic) *management should be based on the parameters obtained without CVVH*”
						Drop in CI and GEDVI		
						Increase in EVLWI		
						Drop in CI and GEDVI and increases in EVLWI are more pronounced in faulty catheter position		
Heise et al. [12]	32	CVVHF	SVC	SVC, IVC	183 ± 35	CO under *running CRRT* lower than at *interrupted CRRT*, but clinically negligible But: exclusion of the first measurement after switching CRRT (on/off) reduces these differences	–**	CO measurements after CRRT stopped/started when blood temp. has reached steady state; exclude first measurement after interruption/continuation of CRRT
Dufour et al. [11]	69	CVVHF	SVC (internal jugular vein)	SVC (7 patient), IVC (62 patients)	250–350	Independently of the catheter tip position, no significant difference in CI and GEDVI in CVVH performed with high blood pump flow up to 350 mL/min detected when the blood pump was stopped Significant, but clinically not relevant decrease in EVLWI when blood pump was turned off in patients with a femoral dialysis catheter	–**	Blood pump should not be stopped
Pathil et al. [7]	30	SLED	IVC, SVC, separate, not used for hemodialysis	SVC, IVC,	–	*During RRT* Significant, but “*narrow*” and therefore “*possibly clinically acceptable”* decrease in CI Significant decrease in GEDVI Discrete, not statistically significant, but “*potentially clinically relevant*” reduce in EVLWI	N.d.	Carefully interpret measurements during RRT
Huber et al. [3]	32	SLED	SVC, IVC (different to dialysis cath.)	SVC, IVC	150	*After connection to RRT* No significant changes in CI *After disconnection of RRT* Significant increase in CI, CPI and GEDVI No significant difference in EVLWI	N.d.	TPTD is accurate despite ongoing RRT
Own study	24	SLED	SVC	SVC, IVC	180 ± 37	*Pausing of RRT* Significantly increased CI and GEDVI Significantly decreased EVLWI and SVRI Independent from dialysis catheter position Independent from blood flow rate No effect, if first measurement within a series was rejected	N.d.	SLED should not be paused for TPTD

*Influenced TPTD, **did not influence TPTD

At first, some authors doubted whether TPTD at ongoing RRT delivers valid data [4–8]. They detected significant changes in the CO [5]/CI [4, 6–8], ITBVI [4, 5]/GEDVI [6, 8] and EVLWI [5, 6, 8] at ongoing RRT depending on the dialysis catheter location (catheter behind temperature detector tip versus between bolus injection and temperature detector) and therefore did not advise to measure during ongoing RRT [4–6, 8] or at least recommend a careful interpretation of these data [7].

In contrast, other authors [10–12] systematically evaluated the optimal time point of TPTD measurements and detected significant changes between measurements at ongoing versus paused/disconnected/no RRT. In line with our results, these authors concluded that small changes in measurements are clinically negligible and deemed TPTD measurements at ongoing RRT to be valid not justifying pausing the dialysis system during measurement. In one of these studies [12], a quintuplicate measurement was applied, and the authors recommended: “If system should be paused, at least reject the first measurement or wait until blood temperature has normalized.” In our study, we could also show that the variability for the CI and GEDVI of the triplicate measurements at paused RRT was higher than ongoing RRT. We calculated the mean differences between the first and the two other measurements, but did not find a meaningful difference between the first and consecutive measurements. Nevertheless, we also detected that the change from running to paused RRT influenced the variability of TPTD results, and therefore, time should be given allowing to reach a steady state before TPTD measurements after pausing/stopping the RRT.

**Fig. 3:**
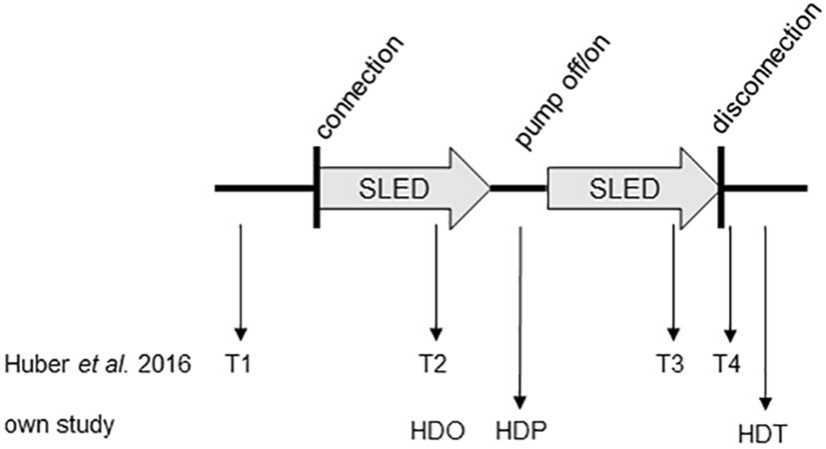
Comparison of the measurement time points to investigate the influence of ongoing SLED on TPTD results between Huber et al. 2016 and our study. Huber et al. 2016 performed their TPTD measurements before connection of the pump (T1), during SLED with pump on (T2), during SLED with pump on after pausing the RRT (T3) and after disconnection (T4). We performed our measurements during SLED (HDO), at paused SLED (pump off/on, HDP) and after termination (HDT)

In all these studies, a continuous RRT (CVVH(D)) was applied, whereas in the present study, the effect of SLED on TPTD was evaluated. Due to the higher transfer volume of cold substitute (9 L/h of dialysate in SLED versus 2 L/h in CVVH) as compared to other RRT procedures, one would expect more pronounced temperature changes that might interfere with TPTD measurements. In addition, there is no active heating in the SLED, which leads to the cooling of the dialysate by 0.5 °C/h, implying that further temperature fluctuations are difficult to calculate. We also investigated the optimal time to recalibrate the system while SLED is already in progress. To the best of our knowledge, only two studies also systematically addressed the reliability of TPTD during SLED. Pathil et al. [7] measured every 8 h during phases of hemodynamic stability without hemodialysis and immediately after onset of SLED. They detected a significant, but clinically acceptable decrease in CI, a significant decrease in GEDVI and a discrete reduction in EVLWI and concluded that measurements during SLED should be carefully interpreted when relying solely on these. They compared measurements before and immediately after onset of the SLED, but this is not comparable to our approach and does not answer if recalibration of TPTD should be performed at ongoing or paused SLED. Huber et al. [3] performed TPTD immediately before and 5 min after connection to SLED, as well as immediately before and after disconnection of SLED and re-transfusion, whereas we measured while ongoing, at interrupted and after terminated SLED and re-transfusion of blood (Fig. 3). Comparable to our findings, TPTD results in these studies seem relatively independent of the presence of a running RRT in a steady state, despite the presumed higher temperature fluctuation using SLED. Accordingly, Huber et al. concluded that TPTD is feasible at ongoing RRT, but, in contrast to our study, they did not separately evaluate if pausing has an influence on TPTD. This aspect had already been studied by Sakka, Dufour and Heise [3, 10–12], who found no [11] or a clinically irrelevant [10, 12] difference between running RRT and paused RRT, although data are only available considering CVVH(D) techniques. Our data add some further knowledge also considering effects of pausing SLED, as an increasingly used RRT technique, on TPTD measurements.

Huber’s and our study show that the changes in CI found during RRT did not influence the TPTD results in a clinically relevant manner. However, in theory the extracorporeal blood flow might lead to a loss of the injected bolus and thus may falsify the determination of the pumped blood volume of the heart by time, defined as CO or CI [per body surface area (BSA)]. The higher the blood flow, the higher (in theory) the loss of the bolus volume, thus leading to an overestimation of the CO up to a maximum of 300%. A ratio (blood flow to CO) below 0.5 seems not to affect TPTD accuracy [18]. In our study, there was a detectable, but clinically not meaningful correlation between the blood flow rate and the CI, considering different settings (HDO, HDP, HDT). Additionally, we calculated the ratio between the blood flow and the CO and compared it with the study of Sakka, Dufour and Heise, using CVVHF (Table 5). The calculated ratios were between 0.01 and 0.06, far below 0.5, and therefore indicating that the accuracy of TPTD was not affected by different blood flow rates. The blood flow rate in our patients was relatively high in combination with a comparably low CI, and though the blood flow/CO ratio was relatively high, we did not detect a negative impact on TPTD.

**Table 5:** Calculation of the ratio between the blood flow and CO

with RRT	Blood flow (L/min)	CI (L/min/m^2^)	CO (L/min) [with BSA = 1.73 m^2^]	Ratio blood flow/CO
Sakka et al. [10]	0.08–0.15	3.9	6.75	0.01–0.02
Dufour et al. [11]	0.25–0.35	3.49	6.04	0.04–0.06
Heise et al. [12]	0.15–0.22	–	6.79 (running/baseline)	0.02–0.03
Own study	0.18	3.1	5.36	0.03

The location of the dialysis catheter in relation to the CVC, via which the cold bolus is applied, might also influence the TPTD measurements [10, 14–16]. In the situation when TPTD is performed in combination with RRT, the proximity of the dialysis catheter to the CVC suggests that this might falsify TPTD results by immediate extraction of the cold bolus. Sakka et al. 2007 described no evidence that this effect relevantly influenced their results. They measured at low blood flow rates, and they did not see any influence of the dialysis catheter position either whether it was placed in the same vessel as the CVC or not [10]. Dufour et al. reported that the paused RRT significantly increased the blood temperature but did not influence the temperature difference between the blood in the SVC and the femoral artery. The authors postulated that the low extracorporeal blood flow rate did not induce a significant alteration in the thermal effect of the cold boluses [11]. Martinez-Simon et al. detected a two-peak temperature curve in the femoral artery during RRT after injection of the cold bolus. They inserted the bolus into the third lumen of a dialysis catheter and did not use a separate central line for PiCCO™. They concluded that the bolus might have directly been aspirated by the hemofiltration flank and therefore been re-injected later [4].

In our study, the cold bolus was injected into a separate CVC avoiding a possible aspiration and we could demonstrate that the location of the dialysis catheter did not relevantly influence the TPTD measurements. Although the number of patients with the catheter placed in the SVC is more than twice compared to the number of patients with the catheter placed in the IVC in our study, the results are statistically tested for equivalence and reliability. These results are in line with those of the studies of Sakka et al. and Dufour et al; both did not detect a difference in TPTD measurements when the dialysis catheter tip is placed in the SVC as compared to IVC [10, 11]. Van Craenenbroeck et al. [8] described similar results, but observed a more pronounced difference between TPTD results with and without RRT when the PiCCO™ catheter was placed in the IVC.

There are some important limitations of our study. First, we did not validate our TPTD measurements against another method to set this as a gold standard. In addition, we did not validate TPTD-derived CI with another method for the measurement of cardiac output, which would not be affected by SLED. Using echocardiography of aortic blood flow as an example would not likely be affected by the temperature or flow rate of blood returning from the SLED device into the vena cava or the right atrium. Hence, we are unable to conclude if a systematic bias introduced by SLED favors either paused, ongoing or terminated SLED during TPTD measurement. Second, we did not measure the body and blood temperature. A limitation of TPTD measuring during RRT could be a decreased accuracy of the measurements as they are highly relying on the blood volume and temperature. Even small changes in the indicator volume might falsify the results of the hemodynamic monitoring. This liability of measurement accuracy on minimal variations in the blood temperature implies to test variations in the indicator bolus such as cooled versus room temperature saline or the replacement of the saline with a lithium dilution. Since neither the body nor the blood temperature was evaluated before bolus injection or at the time of pausing and restarting the dialysis, these effects on TPTD measurements could not be tested. It would be interesting to evaluate if a longer waiting period before measurement may improve the accuracy of measurements compared to the presumably true value obtained after stopping the dialysis. Furthermore, our conclusions might only apply to patients with the CVC for TPTD measurements placed in the SVC. Results could be different when the catheter is inserted in the femoral vein, especially in cases with abdominal hypertension. Finally, the data are of observational nature with a small number of patients, in particular concerning the small number of patients with location of the dialysis catheter in the IVC, which resulted from the fact that the femoral access route is not common in our clinic, and independently thereof repeated measurements within many patients. As this study tried to mirror every day clinical practice and as it is an exploratory study, we aimed to investigate if larger (clinically relevant) differences between the TPTD measurements are to be expected for ongoing versus interrupted SLED. From this, we did no sample size calculation a priori. Since there was a clear statistical difference between measurement conditions (even in this small sample size), a sample size calculation is not necessary for this exploratory study.

## Conclusions

TPTD measurements are not clinically meaningful influenced by ongoing SLED, regardless of blood flow and location of the dialysis catheter. However, starting or stopping the dialysis temporarily affected TPTD results. In a real-life scenario, SLED is running for up to 12 h and a TPTD measurement/recalibration of the PiCCO™-system needs to be performed regularly or in case of hemodynamic deterioration during this process. The easiest and most efficient way to perform these measurements in clinical routine would be during ongoing SLED, or, if this would deliver falsified results, at paused SLED. We have shown that measurements at ongoing SLED deliver accurate results, and as this is the most convenient way to perform routine measurements, our data therefore suggest that the dialysis system needs not to be stopped for the TPTD measurements.

## Abbreviations

AKI: acute kidney injury
BSA: body surface area
CE: coefficient of error
CI: cardiac index
CV: coefficient of variation
CVC: central venous catheter
CVVH(D/F): continuous veno-venous hemofiltration/hemodialysis/hemodiafiltration
EVLWI: extravascular lung water index
GEDVI: global end-diastolic volume index
HD: hemodialysis
HDO: ongoing hemodialysis
HDP: paused hemodialysis
HDT: terminated hemodialysis
IVC: inferior vena cava
LSC: least significant change
MAP: mean arterial pressure
MOF: multiple organ failure
RRT: renal replacement therapy
SD: standard deviation
SLED: sustained low efficiency dialysis
SVC: superior vena cava
SVRI: systemic vascular resistance index
TPTD: transpulmonary thermodilution
